# The Pattern of Valvular Heart Diseases in India During Pregnancy and Its Outcomes

**DOI:** 10.7759/cureus.16394

**Published:** 2021-07-14

**Authors:** Anupama Kumari, Kamlesh Kumar, Abhay Kumar Sinha

**Affiliations:** 1 Obstetrics and Gynaecology, Patna Medical College, Patna, IND; 2 Cardiology, Jawaharlal Institute of Postgraduate Medical Education and Research, Puducherry, IND; 3 Internal Medicine, Patna Medical College, Patna, IND

**Keywords:** valvular heart disease, india, pregnancy, rheumatic, mitral stenosis, aortic stenosis, maternal outcomes, perinatal outcomes

## Abstract

Introduction: The incidence of rheumatic heart disease is very high in India. The data on the pattern of valvular heart diseases during pregnancy and its outcomes is very scarce. Again, the data in the Indian scenario, the differences in outcomes between different grades of valvular heart diseases and its impact on pregnancy outcomes is very less. We planned to study the different patterns of valvular heart diseases during pregnancy and their outcomes with respect to cardiac complication and perinatal outcomes.

Materials and methods: It was a hospital-based prospective observational study. We recruited 71 patients after taking written informed consent. All patients were with term gestation and valvular heart diseases. We did 2D echocardiography to analyze the valve lesion and assess the valve lesion with its maternal and perinatal outcomes.

Results: The mean age of participants in the study was 27 + 5.2 years. A total of 54 patients (76.1%) were less than 30 years and 17 (23.9%) were more than 30 years of age. Six patients (8.5%) presented with New York Heart Association (NYHA) class I, 39 patients (54.9%) presented with NYHA class II, 25 patients (35.2%) presented with NYHA class III and one patient (1.4%) presented with NYHA class IV. The most common etiology of valvular heart diseases was found to be rheumatic in 62 patients (87.3%). The most common valve involved was the mitral valve (69%). New-onset atrial fibrillation (AF) was reported in 26.8% patients and pulmonary edema developed in 15.5% patients. Live birth was observed in 66 patients (93%) compared to stillbirth reported in five patients (7%).

Conclusion: No significant difference in maternal and perinatal outcomes between moderate and severe grades of different valvular heart diseases.

## Introduction

Pre-existing cardiac diseases is a major contributor to maternal mortality and mortality, particularly in low- and middle-income countries. The prevalence of rheumatic heart disease, in India, is high accounting for 40%-50% of all cardiac diseases during pregnancy [1]. The incidence of maternal mortality rate has quite decreased with respect to obstetrics condition but remains high in cardiac diseases especially rheumatic heart diseases [2]. The incidence of heart diseases is high at around 0.9%-3.1% [3]. The physiological and hemodynamic changes also pose risks to maternal health and influence maternal morbidity and mortality. The systemic vascular resistance decreases by the first trimester with cardiac output increases especially in the middle trimester [4].

As per modified WHO classification for maternal cardiovascular risk, severe mitral stenosis (MS) and severe aortic stenosis (AS) are classified as risk class IV but less data are available regarding the pregnancy outcomes in cases of a moderate grade of both MS and AS. The data with regard to the Indian population are even scarce. The different patterns of valvular heart diseases (VHD) apart from rheumatic heart diseases are mitral valve prolapse, bicuspid aortic valve (BCAV) and secondary to cardiomyopathy. Their prevalence and their association with maternal complications and perinatal outcomes are also less known to the Indian population. Prevalence of cardiac complications like new-onset arrhythmia, heart failure and deterioration of functional capacity in a moderate grade of MS and AS are also less described with respect to Indian data. So, in order to assess the risk associated with different VHD and their impact on outcomes in terms of maternal and fetal mortality, we carried out a prospective study to determine the pattern of VHD among women coming to labor room.

## Materials and methods

The study was conducted at Patna Medical College and hospital. It was a hospital-based prospective observational study. This study was approved by Institutional Ethics Committee for Research Studies, Patna Medical College (PMC/IEC/2019/111). We recruited 71 patients in our study who were more than 18 years of age after written informed consent. All patients with term gestation (>37 weeks) were included in the study. Those with mild VHD were excluded from the study. Patients with poor compliance from medication were also excluded from the study. All pregnant women with term gestation and VHD were assessed in the labor room.

Baseline characteristics were noted with respect to age, New York Heart Association (NYHA) class and severity of VHD.

A 2D echocardiography with color doppler was done to analyze the valve lesion with respect to location, etiology and severity. The different pattern of VHD included were rheumatic, mitral valve prolapse and BCAV. The lesions were graded into mild, moderate and severe. The mild variants of valvular lesions were excluded from the study.

Cardiac complications were observed in terms of deterioration of functional class, new onset of atrial fibrillation (AF) and pulmonary edema during labor. Cardiac complications during labor were measured in terms of deterioration of functional class, new-onset AF and development of pulmonary edema. The fetal complication was measured as perinatal outcomes in form of either live birth or stillbirth.

Each of the valvular lesions was tested for its association with maternal complication and perinatal outcomes developed during labor. Again, moderate and severe grades of each valvular lesion were compared and analyzed for its association with the cardiac complication and perinatal outcomes developed during labor.

Statistical significance was calculated at 95% confidence interval (p-value<0.05). Association between categorical variables was calculated with chi-square test, Fisher exact test and ANOVA. Results were analyzed with IBM SPSS version 20 (IBM Corp., Armonk, NY, USA).

## Results

A total of 71 patients were enrolled in the study. The mean age of participants in the study was 27 + 5.2 years. A total of 54 patients (76.1%) were less than 30 years and 17 (23.9%) were more than 30 years of age. Six patients (8.5%) presented with NYHA class I, 39 patients (54.9%) presented with NYHA class II, 25 patients (35.2%) presented with NYHA class III and one patient (1.4%) presented with NYHA class IV. Baseline characteristics of patients are tabulated in Table 1.

**Table 1:** Baseline characteristics (n=71).

Maternal characteristics	Number (%)
Mean age (SD)	27+5.2 years
<30 years	54 (76.1%)
>30 years	17 (23.9%)
NYHA functional class	
Class I	6 (8.5%)
Class II	39 (54.9%)
Class III	25 (35.2%)
Class IV	1 (1.4%)

The most common etiology of VHD was found to be rheumatic in 62 patients (87.3%). Mitral valve prolapse was found in seven patients (9.9%) and BCAV was found only in two patients (2.8%).

The most common valve involved was mitral valve (69%). Moderate MS was present in 13 patients (18.3%). Severe MS was present in 36 patients (50.7%). The mitral regurgitation (MR) was present in 25 (35.2%) of patients. Moderate MR was present in 18.3% of patients and severe MR was present in 16.9% of patients. The AS was present in seven patients (9.8%). Moderate and severe AS were present in 4.2% and 5.6% of patients, respectively. The aortic regurgitation (AR) was present in 12 (17%) of patients. Moderate AR and severe AR were present in 8.4% each.

A maximum of 39 patients (54.9%) were in NYHA II class, 25 patients (35.2%) were in NYHA III class, six patients (8.5%) in NYHA class I and one patient (1.4%) in class IV.

Cardiac lesions are tabulated in Table 2.

**Table 2:** Types of cardiac lesions (n=71).

Rheumatic heart disease	62 (87.3%)
Severe mitral stenosis	36 (50.7%)
Moderate mitral stenosis	13 (18.3%)
Mitral valve prolapse (MVP)	7 (9.9%)
Mitral regurgitation (Rheumatic plus MVP)	25 (35.2%)
Moderate mitral regurgitation	13 (18.3%)
Severe mitral regurgitation	12 (16.9%)
Bicuspid aortic valve (BCAV)	2 (2.8%)
Aortic stenosis (BCAV plus Rheumatic)	7 (9.8%)
Moderate aortic stenosis	3 (4.2%)
Severe aortic stenosis	4 (5.6%)
Aortic regurgitation (BCAV plus Rheumatic)	12 (17%)
Moderate aortic regurgitation	6 (8.4%)
Severe aortic regurgitation	6 (8.4%)

Cardiac complications during labor were measured in terms of deterioration of functional class, new-onset AF and development of pulmonary edema. The fetal complication was measured as perinatal outcomes in form of either live birth or stillbirth.

Deterioration of functional class was observed in 22 patients (31%). New-onset AF was reported in 19 patients (26.8%). Pulmonary edema developed in 11 patients (15.5%). Live birth was observed in 66 patients (93%) compares to stillbirth reported in 5 patients (7%). Cardiac complication and perinatal outcomes are tabulated in Table 3.

**Table 3:** Cardiac complications and perinatal outcomes in pregnant women during delivery (n=71).

Cardiac complication	
New onset atrial fibrillation	19 (26.8%)
Pulmonary edema	11 (15.5%)
Deterioration of functional class	22 (31%)
Perinatal outcomes	
Live birth	66 (93%)
Still birth	5 (7%)

In our study, more than 50% of patients delivered through cesarean section. The decision of mode of delivery was as per cardiac or obstetric indications. Forty-five patients (63.4%) delivered through cesarean section compare to 26 patients who delivered normal vaginal delivery.

There was an association between the severity of MS and the mode of delivery. Severe MS were delivered more through cesarean section (either cardiac indication or obstetrics indication) compared to moderate MS (p<0.01). There was no difference found between severe MS and moderate MS for the onset of pulmonary edema during labor (p>0.18). There was no difference found between severe MS and moderate MS for the new-onset AF during the peripartum period (p>0.22). There was no difference found between severe MS and moderate MS for the perinatal outcome (in terms of live birth or stillbirth) during the peripartum period (p>0.78).

There was an association between the severity of MR and deterioration in functional class during the peripartum period. Deterioration in functional class was more observed with severe MR compared to moderate MR (p<0.03). There was no association between the severity of MR and the development of pulmonary edema during labor. There was no association between the severity of MR and mode of delivery (p>0.82). There was no difference found between severe MR and moderate MR for the new-onset AF during the peripartum period (p>0.57). There was no difference found between severe MR and moderate MR for the perinatal outcome (in terms of live birth or stillbirth) during the peripartum period (p>0.28).

There was no association between the severity of AS and mode of delivery during labor. There was no association between severity of AS and deterioration in functional class during peripartum period. (p>0.35). There was no difference found between severe AS and moderate AS for the new-onset AF during the peripartum period (p>0.35). There was no difference found between severe AS and moderate AS for the perinatal outcome (in terms of live birth or still birth) during the peripartum period (p>0.35).

There was no association between the severity of AR and deterioration in functional class during the peripartum period (p<0.51). There was no association between the severity of AR and the development of pulmonary edema during labor (p>0.12). There was no association between the severity of AR and mode of delivery (p>0.08). There was an association found between the severity of AR and for the new-onset AF during the peripartum period. It was more observed with moderate AR with other coexistent lesions (p>0.04).

## Discussion

In our study, we determined the severity of different VHD and assess their outcomes on maternal complication and fetal complication. Heart diseases continue to be a major risk factor for pregnancy outcomes in terms of maternal morbidity and mortality [5]. In a study by Hagen et al. [6], they found rheumatic origin being the most common etiology of VHD during pregnancy. In our study also, rheumatic origin of VHD was seen in 87.3% of patients. In our study, the mean age of participants was 27 + 5.2 years. 76.1% were young and less than 30 years old.

Maternal complication during labor includes heart failure, pulmonary edema, new-onset AF and other thromboembolic complication. This complication contributes maximally to maternal and fetal morbidity and mortality [7]. In our study, maximum patients (54.9%) presented with NYHA class II. New-onset AF was reported in 26.8% patients and pulmonary edema developed in 15.5% patients.

As per the modified WHO classification of maternal cardiovascular risk, patients with symptomatic severe MS and AS are at extreme risk for maternal morbidity and mortality, and pregnancy is contraindicated [8]. But in India where the incidence of rheumatic heart diseases is so high especially in females, the numbers of pregnant females with severe MS and AS coming to labor room are also high. Even if pregnancy is continued upon, intensive specialist cardiac and obstetric is required throughout pregnancy and puerperium.

In a study by Desai et al. [9], MS was found to be most common lesion during pregnancy. In our study, MS was the most common lesion found in 69% of patients. Patients with severe MS are more prone to develop a cardiovascular complication in terms of functional class deterioration and are at significant risk for cardiac decompensation [10]. In our study, deterioration of functional class was observed in 31% of patients during labor. In our study, although severe MS delivered more through cesarean section compared to moderate MS, there was no difference in other outcomes like for the development of pulmonary edema during labor (p>0.18), for the new-onset AF during the peripartum period (p>0.22), and for the perinatal outcome (in terms of live birth or stillbirth) during the peripartum period (p>0.78).

MR is usually well tolerated during pregnancy because of lowered systemic vascular resistance. AR is also well tolerated in NYHA I and II forms [11]. In our study comparing severe MR and moderate MR, there was no significant association between the development of pulmonary edema and severe MR. In other outcomes also, like new-onset AF (p>0.57) and perinatal outcomes (p>0.28), there were no differences found between severe MR and moderate MR.

With severe AS, there were no significant differences among different outcomes like pulmonary edema, perinatal outcome and new-onset AF between moderate and severe AS [12].

With AR, there were no significant differences among different outcomes between moderate and severe AR except association with the development of pulmonary edema, which happened more with moderate AR. The association of moderate AR with pulmonary edema can probably be explained by the presence of coexistent lesions. In the majority of cases the moderate AR was coexistent with either severe MS or other significant grades of valvular lesions [13].

## Conclusions

During pregnancy, there is a marked physiological increase in blood volume and then a corresponding increase in heart rate and cardiac output. Such high volume is poorly tolerated with patients with VHD especially stenotic lesions. And hence such lesions are at significant risk of developing arrhythmia, pulmonary edema and heart failure.

Although severe symptomatic MS and severe AS are potential risk factors for pregnancy outcomes and as per modified WHO classification of maternal cardiovascular risk, both are WHO risk class IV, but their moderate grade of valvular lesions (moderate MS and moderate AS) also poses a significant risk to maternal morbidity and mortality and influence perinatal outcomes. VHD with moderate AS and moderate MS imposes a significant risk to maternal morbidity and severe morbidity. If pregnancy is decided upon with such valvular lesions, intensive specialist cardiac and obstetric monitoring are required throughout pregnancy and puerperium.
